# A meta-analysis of the relationship between anxiety and non-suicidal self-injury based on knowledge graphs

**DOI:** 10.3389/fpsyt.2024.1493823

**Published:** 2025-01-06

**Authors:** Jieyao Shi, Pan Gao, Bingqian Zhou, Zhisheng Huang

**Affiliations:** ^1^ Suzhou Vocational University, Jiangsu, China; ^2^ Mental Health Center affiliated to Tongji University, Shanghai, China

**Keywords:** non-suicidal self-injury, anxiety, meta-analysis, knowledge graph, SPARQL

## Abstract

**Objective:**

The existing research on the relationship between anxiety and non-suicidal self-injury (NSSI) is inconsistent, and there is no systematic review on this area. This study aims to explore the relationship between anxiety and NSSI, in order to provide evidence-based medicine evidence for the early identification of preventable occurrence factors of NSSI.

**Methods:**

The semantic query (i.e. SPARQL) method was used to retrieve the anxiety- related literature on the Knowledge graph of NSSI, which consist of the metadata and semantic annotation data of English literature related to non-suicidal self-injury in PubMed by June 2023. Two researchers strictly followed the inclusion and exclusion criteria for independent literature screening. After evaluating the quality of the included studies, the selected data was subjected to meta-analysis using RevMan5.3 software.

**Results:**

A total of 14 studies met the inclusion criteria of the meta-analysis, including 44064 subjects. The results showed that the proportion of anxiety in the NSSI group was significantly higher than that in the non-NSSI group, and the difference between the groups was statistically significant (OR=3.60, 95% Cl=2.08-6.22, p<0.01).

**Conclusion:**

There is a significant correlation between anxiety and NSSI, which is a possible risk factor for NSSI. However, due to limitations of the design type, quantity, and quality of the included study, further research is needed on the causal relationship between anxiety and NSSI. Furthermore, we show that using knowledge graphs is an effective approach to retrieve literature for meta-analysis.

## 1 Introduction

Non-suicidal self-injury (NSSI) is a recurring behavior of directly and intentionally injuring one’s own organs and tissues without the purpose of death and does not result in death (1). Currently, NSSI is receiving more and more attention worldwide due to its high detection rate, high risk, and high recurrence, and has become an important public health problem that cannot be ignored. According to statistics, about 14% to 15% of adolescents worldwide have engaged at least one NSSI behavior (2), and the lifetime prevalence of NSSI in adults and adolescents is about 5.5% and 17.2%, respectively (3). NSSI has been studied as a separate clinical disorder in the Diagnostic and Statistical Manual of Mental Disorders, 5th edition (DSM-V) in the United States (4). It has been shown that adult and adolescent populations with NSSI have a higher risk of suicide than their non-NSSI counterparts (5), so NSSI is an important risk factor for predicting suicide (6–8). NSSI not only causes serious physical and psychological harm to itself and suffering to the family, but also adds a huge economic burden to society (9). Therefore, early identification and screening to NSSI preventable risk factors are of great importance.

In the last two decades or so, research has increasingly focused on identifying risk factors that may increase the occurrence or persistence of NSSI (10, 11), with considerable attention paid to emotion-related factors (12). Studies have shown that stressful events have an indirect effect on NSSI through anxiety symptoms (13, 14), and that NSSI becomes a coping strategy to regulate negative emotions including anxiety and depressive symptoms (11), and that NSSI serves as a way of avoiding or distracting from anxiety arousal or worrying about the possible adverse consequences of such arousal (15), which can reduce internal suffering and keep away from negative emotional states such as anger, sadness, numbness, or shame and other negative emotional states (16, 17). Many scholars have proposed various theoretical models to explain the mechanism of NSSI behavior on the basis of previous research on the influencing factors of NSSI, such as Nock et al.’s four-function model (18) and Hasking et al.’s cognitive-emotional model (19), Chapman et al.’s experiential avoidance model (20), etc., all show that emotion regulation is an important mechanism of NSSI behavior. Anxiety as a major emotional factor deserves in-depth study.

Although more studies have shown the correlation between anxiety and NSSI, the results of existing studies are not consistent. It was found that NSSI patients showed higher levels of anxiety than those without NSSI behaviors, and anxiety symptoms were positively correlated with NSSI (14, 21); another longitudinal study found that anxiety or stress was correlated with NSSI (22); however, Loh et al.’s study showed the opposite result, anxiety was negatively correlated with NSSI (23); and McCoy et al. found that anxiety did not differ significantly between the NSSI and non-NSSI groups (24). As the results of each individual study are often inconsistent due to small sample sizes or methodological limitations, this inconsistency makes the use of individual study results as the basis for decision-making restrictive and may lead to inaccurate or misleading conclusions. Meta-analysis expands the sample content by synthesizing multiple small samples to improve the test efficacy; a comprehensive synthesis and quantification of controversial or even contradictory studies of the same kind can strengthen the level of evidence, make more accurate estimation of the effects, and lead to more definitive and comprehensive conclusions that will provide valuable references for the prediction of the occurrence and maintenance of NSSIs.

## 2 Materials and methods

### 2.1 Search strategy

Knowledge Graphs are widely used to make a large scale semantic network consisting of entities and concepts as well as the semantic relationships among them, using representation languages such as RDF and RDF Schema (25). Such knowledge graphs are used in the construction of many knowledge-based applications in medicine, such as extracting information from patient records (26), personalized medicine (27), and many others.

We have constructed Knowledge Graphs of NSSI (called NSSIKG for short). The NSSIKG integrates a variety of knowledge resources related to NSSI, including medical literature, and clinical ontology knowledge bases such as SNOMED CT in clinical medical concept terminology, etc. By constructing Knowledge Graphs of NSSI, comprehensive knowledge can be effectively transformed into well-structured knowledge.

**Figure 1:**
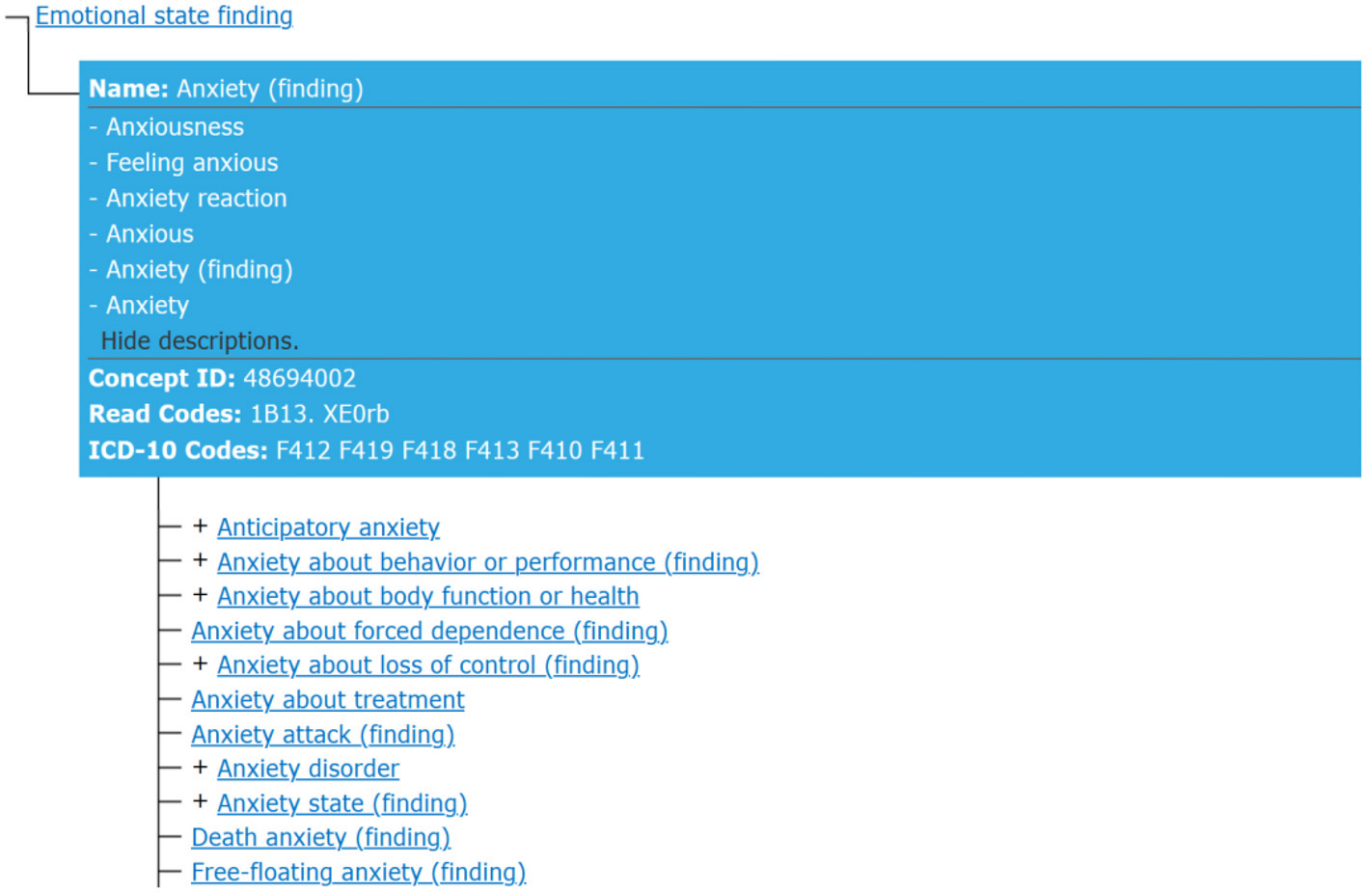
The top-level conceptual ID screening.

**Figure 2:**
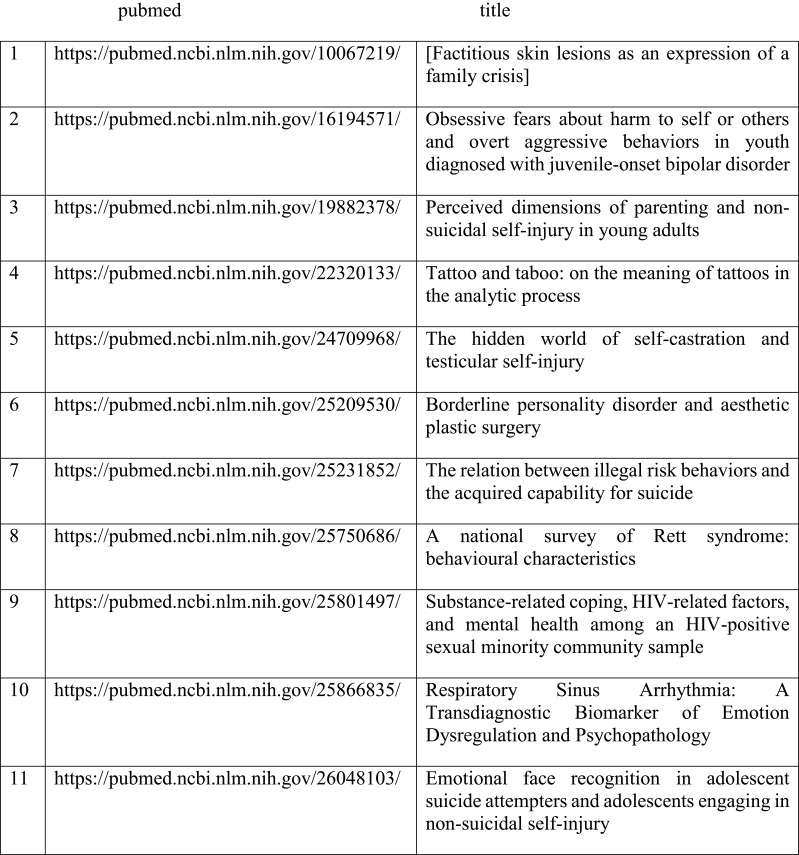
Some of the results of the retrieval.

By analyzing the medical conceptual terminology SNOMED CT, we identify the top-level conceptual ID for anxiety is 48694002 (see **Figure 1**), which has 283 sub-concept labels such as Acarophobia, Algophobia, Androphobia, Anxiety neurosis, Erotophobia, and many others. Using the traditional key word search method, it is impossible for human to exhaust all possible keywords and their synonyms concerning anxiety and its sub-concepts. Thus, we have to use the knowledge graph approach which contains the domain knowledge of medical terminology and can reason with the concept hierarchy to exhaust all of the sub-concept labels about anxiety for the semantic search. Namely a semantic query was applied to query and reason over Knowledge Graphs of NSSI, which consist of the metadata and semantically annotated data of English-language literature involving NSSI as of June 2023, which are included in PubMed. Furthermore, using concept IDs to search over anxiety-related literature can reduce duplicate conceptual labels (see **Figure 2** for some of the results of the retrieval). After SPARQ search, further retrieve relevant literature was complemented by methods such as literature tracing. The references related anxiety and NSSI listed in the literature by SPARQL retrieval were searched for the original text one by one, and the references in the newly found original text were further tracked to expand the scope of literature information.

**Figure 3:**
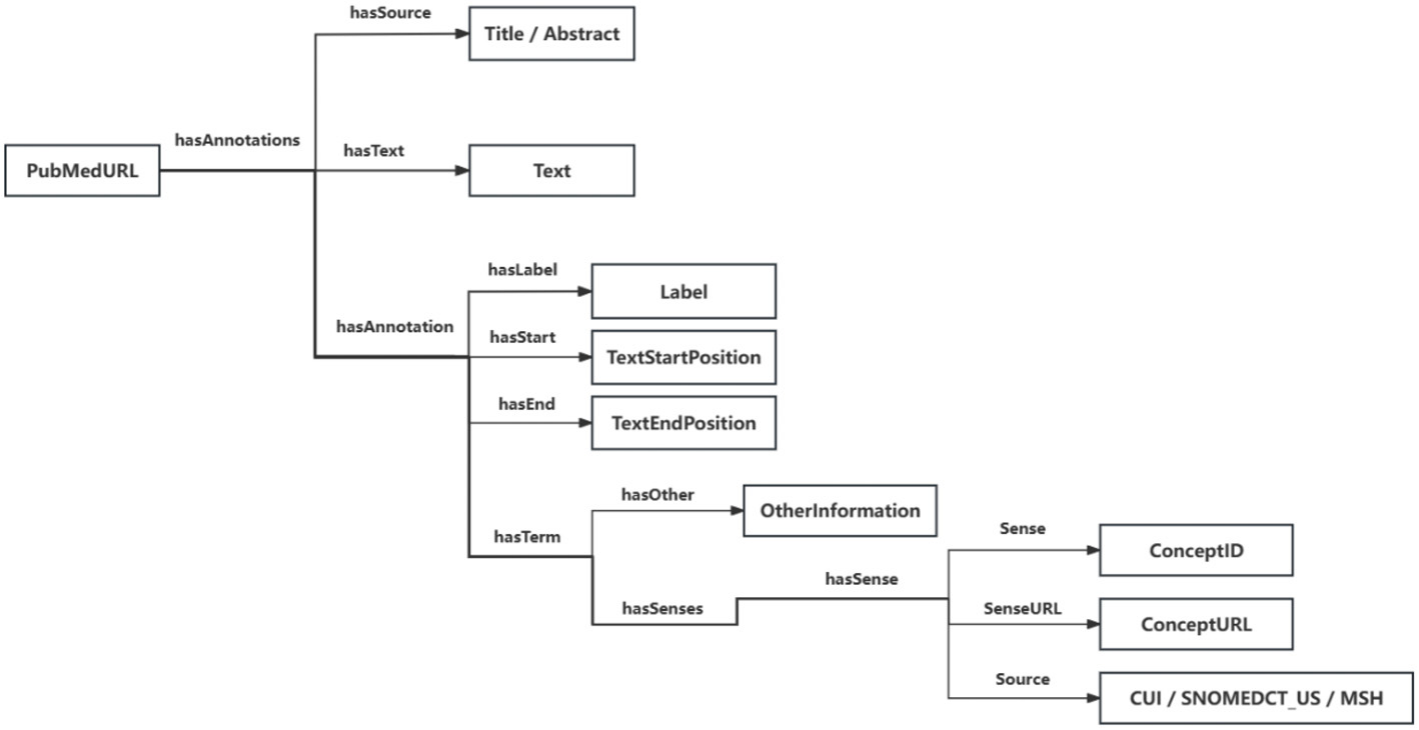
Triple structure.

As mentioned above, Knowledge graphs are knowledge bases which consist of the triple sets (i.e., subject-predicate-object) with the standard data format such as RDF/RDFS for knowledge graph representation. Knowledge graphs support for semantic queries (i.e., SPARQL queries) and reasoning over the semantic annotation data with ontologies/terminologies. The semantic annotation data of the NSSI literature in the NSSI knowledge graph use the following triple structure (see **Figure 3**):

Namely each PubMed paper is made as a set of annotations on its free text of the title or a sentence in the abstract. Each annotation consists of a term with the text (i.e. label) and its corresponding senses of the label. Each sense is identified with its corresponding concepts in terminologies/ontologies such as CUI for the concepts in UMLS, well-knowledge clinical terminology SNOMED CT (US edition), and MSH for the MeSH keyword set for PubMed/Medline. Thus, the corresponding SPARQL queries were.

PREFIX sct: <http://wasp.cs.vu.nl/sct/sct#>

PREFIX drugbank: <https://www.drugbank.ca/drugs/>

PREFIX ztonekg:<http://www.ztonebv.nl/KG#>

PREFIX pubmed:<http://www.ncbi.nlm.nih.gov/pubmed/>

PREFIX rdf: <http://www.w3.org/1999/02/22-rdf-syntax-ns#>

PREFIX rdfs: <http://www.w3.org/2000/01/rdf-schema#>

PREFIX snomed: <http://www.ihtsdo.org/SCT_>

select distinct?pubmed?title

where {

?symptom rdfs:subClassOf* snomed:48694002.

?t1s1 ztonekg:SenseURL ?symptom.

?symptom sct:hasEnglishLabel ?label.

?t1 ztonekg:hasSense ?t1s1.

?s7 ztonekg:hasSenses ?t1.

?s ztonekg:hasTerm ?s7.

?s1 ztonekg:hasAnnotation ?s.

?s1 ztonekg:hasSource “Abstract”.

?s1 ztonekg:hasText ?text.

?pubmed ztonekg:hasAnnotations ?s1.

?pubmed ztonekg:hasAnnotations ?s1b.

?s1b ztonekg:hasText ?title.

?s1b ztonekg:hasSource “Title”.

}

Namely we use the predicate rdfs:subClassOf* to reasoning with the concept hierarchy over anxiety. Furthermore, we use the predicate ztonekg:hasSense and others with matched patterns over the annotation triples to find its corresponding PubMed ID, the abstract text, and the title of the PubMed ID.

The semantic annotation data of the NSSI Knowledge Graphs and the result of the semantic query are available for download at the website https://wasp.cs.vu.nl/NSSI/data/.

### 2.2 Inclusion and exclusion criteria

Inclusion criteria: (1) publicly available, English-language reports of original studies covering NSSI in relation to anxiety; (2) the study design was a cohort or cross-sectional study; and (3) NSSI was defined as intentional self-injurious behavior without suicidal intent.

Exclusion criteria: (1) original design methodology was inaccurate and of low reliability; (2) studies that did not have comprehensive and clear data; (3) literature with errors in statistical calculations or that could not be transformed into ORs and 95% CIs; (4) full text was not available; and (5) repetitively published literature.

### 2.3 Literature screening, data extraction, and quality assessment

The titles and abstracts of the literature were obtained by an independent primary screening search by 2 researchers, the included research literature was identified based on the inclusion and exclusion criteria, and then the full text of the literature obtained from the primary screening was downloaded for evaluation.

Data extraction included literature title, first author, year of publication, country of study, study design, sample source, sample size, and assessment tool.

Evaluation of the quality of the included research literature was performed using the Agency for Healthcare Research and Quality (AHRQ) evaluation tool out of 11 points, and the research literature was categorized as low (0-3), moderate (4-7), and high (8-11) quality according to the scores. Any disagreement in the study was resolved through a third party or discussion.

### 2.4 Statistical analysis

All extracted information was collated and the extracted data were qualitative synthesis by summarizing, comparing, and contrasting, and Meta-analysis was performed using Review Manager 5.0. The 95% confidence interval (95%Cl) and the ratio of ratios (OR) were used as effect sizes, and the combined effect sizes, with a Z-test of P<0.05, were considered statistically significant for the combined results. A heterogeneity test was performed for the included studies, I^2^≦50% and p>0.1, suggesting homogeneity among studies, applying fixed-effects model to analyze; I^2^>50% and p<0.1, suggesting heterogeneity among studies, applying random-effects model to analyze, stability of the results was analyzed using sensitivity analysis, and potential publication bias was evaluated using a funnel plot.

## 3 Results

### 3.1 Results of the literature search

**Figure 4:**
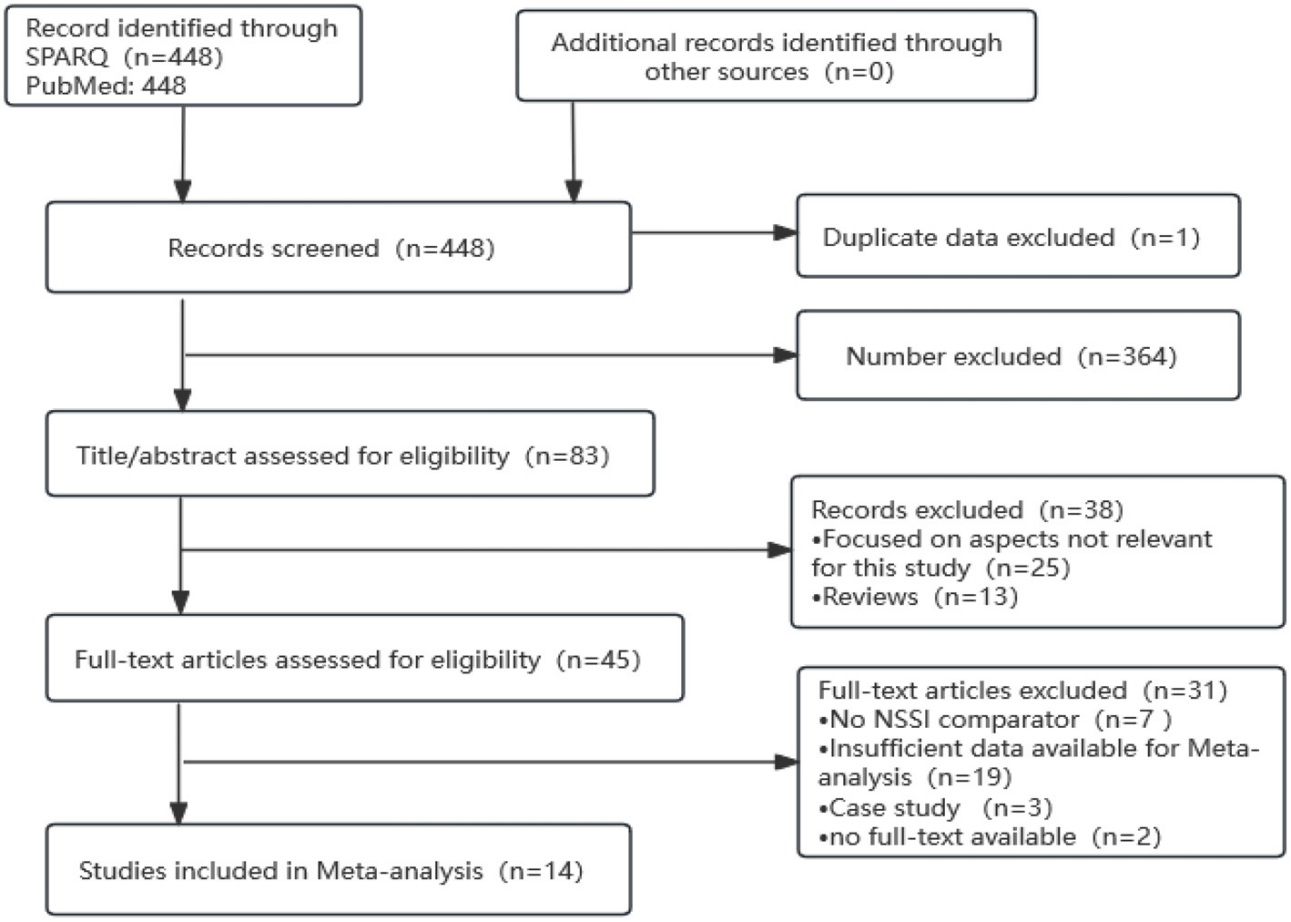
PRISMA flow chart for meta-analysis of publication related to anxiety and non-suicidal self-injury.

The initial search using the knowledge graph generated 448 literature titles, and the retrospective search yielded 0 documents, for a total of 448 documents; after the initial screening, 365 documents were excluded, 38 documents were excluded after reading the titles and abstracts, 31 documents were excluded after reading the full text and through detailed analysis, and finally, 14 (28–41) documents meeting the requirements of this Meta-analysis were included. The literature screening process is shown in **Figure 4**.

### 3.2 Basic characteristics and quality assessment of the included literature

A total of 14 English original research papers were included, including 6 longitudinal studies and 8 cross-sectional studies. The quality evaluation of the included research literature according to the AHRQ evaluation criteria showed that six studies scored between 8 and 11, which were high-quality studies, and eight studies scored between 4 and 7, which were medium-quality literature. The basic characteristics and quality scores of the included data are shown in **Table 1**. Although there were several different types of anxiety, the common characteristic of anxiety was a feeling unease or worry about future events or uncertain situations, and the definitions of anxiety across the included studies were shown in **Table 2**.

**Table 1:** Basic characteristics and quality evaluation of included literature.

Author(s), Year	Country	Sample Source	Age (years)	Study Design	Anxiety Diagnosis	Sample size		Quality Score
						NSSI	non-NSSI	
Chartrand 2012 (28)	American	Community	>18	CS	DSM-IV	85	12985	7
Chartrand 2015 (29)	Canada	Hospital	19~45	CS	DSM-V	230	2380	8
Gollust 2010 (30)	American	University	>18	CS	DSM-IV	199	2644	9
Mars 2014 (31)	England	Community	16	L	DAWBA	569	3905	8
Laporte 2017 (32)	Swedish	Prison	18~25	L	DSM-V	62	208	7
O’Connor 2009 (33)	England	School	15~16	L	HADS	31	469	8
Peters 2018 (34)	England	Community	>16	CS	ICD-10	250	6971	7
Brunner 2007 (35)	Germany	School	<16	CS	CBC	859	4900	7
Coppesmith 2017 (36)	New Zealand	Community	26	L	DSM-IV	191	466	8
Szejko 2019 (37)	Poland	Hospital	5~50	L	DSM-IV	65	100	7
Tuisku 2006 (38)	Finland	Hospital	13~19	L	DSM-IV	24	90	8
Thai 2021 (39)	Vietnam	School	15~18	CS	DASS-21-A	551	704	6
Baralla 2021 (40)	Italy	Hospital	18~68	CS	SAS	45	124	7
Evren 2014 (41)	Turkey	School	16	CS	ESPAD	713	4244	7

Quality Score= Results of literature quality evaluation.

CS, Cross-sectional; L, Longitudinal.

DSM-IV, Diagnostic and Statistical Manual of Mental Disorders, 4th edition; DSM-V, Diagnostic and Statistical Manual of Mental Disorders, 5th edition; DAWBA, the Development and Well-Being Assessment; HADS, The Hospital Anxiety and Depression Scale; ICD-10, The International Statistical Classification of Diseases and Related Health Problems 10th Revision; CBC, The German version of the Youth Self-Report (a self-report version of the Child Behavior Checklist); DASS-21-A, The Depression Anxiety Stress Scale has 21 items that measure symptoms of anxiety (7 items) ; SAS, Zung Self-Rating Anxiety Scale; ESPAD, The European School Survey Project on Alcohol and Other Drugs.

**Table 2:** Definitions of anxiety across the included studies.

Diagnostic Tool	Definition	Effect
DSM-IV	Structured Clinical Interview guides	Relatively accurate morbidity
DSM-V	Structured Clinical Interview guides	Relatively accurate morbidity
DAWBA	Semi-structured interview	Relatively accurate morbidity
HADS	Self-report anxiety subscale	May overestimated morbidity
ICD-10	Fully structured interview	Relatively accurate morbidity
CBC	Self-report anxiety subscale	May overestimated morbidity
DASS-21-A	Self-report anxiety subscale	May overestimated morbidity
SAS	Self-report anxiety scale	May overestimated morbidity
ESPAD	Self-report anxiety subscale	May overestimated morbidity

### 3.3 Meta-analysis results

**Figure 5:**
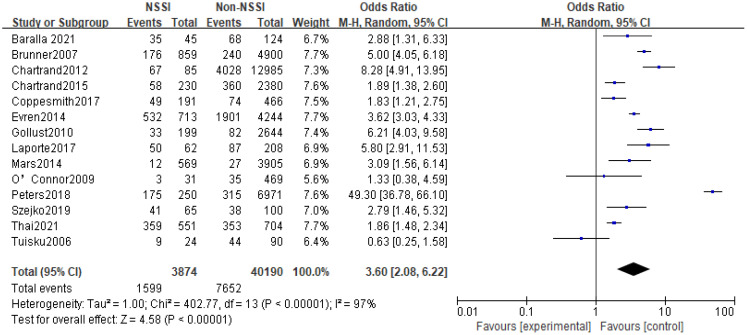
Met-analysis of the relationship between anxiety and NSSI.

All 14 studies reported a correlation between anxiety and NSSI. Of the total study population of 44064, 3874 were in the NSSI group and 40190 were in the non-NSSI group. Heterogeneity between studies was statistically significant (p<0.01, I^2^ = 97%), and Meta-analysis using a random effects model showed that the proportion of anxiety in the NSSI group (41.28%) was higher than that in the non-NSSI group (19.04%), and the difference was statistically significant (OR=3.60, 95% Cl=2.08-6.22, p<0.01), see **Figure 5**.

### 3.4 Analysis of subgroup results

**Figure 6:**
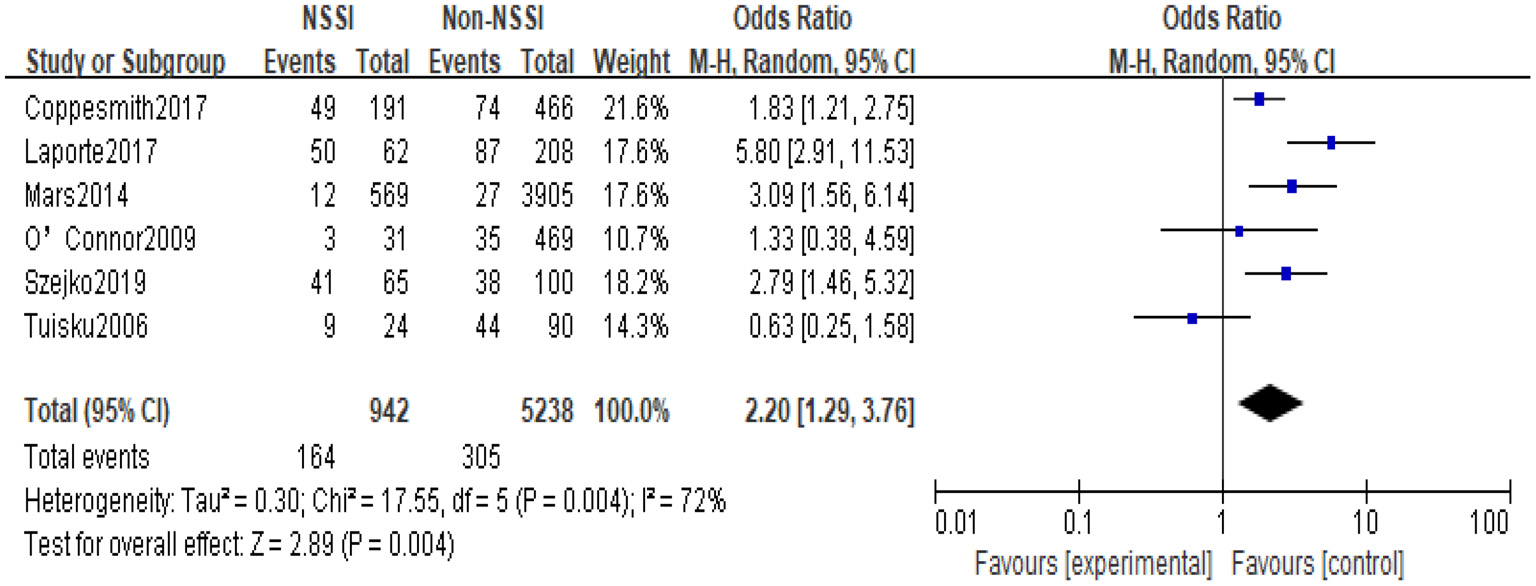
Results of subgroup analysis in the cohort group.

**Figure 7:**
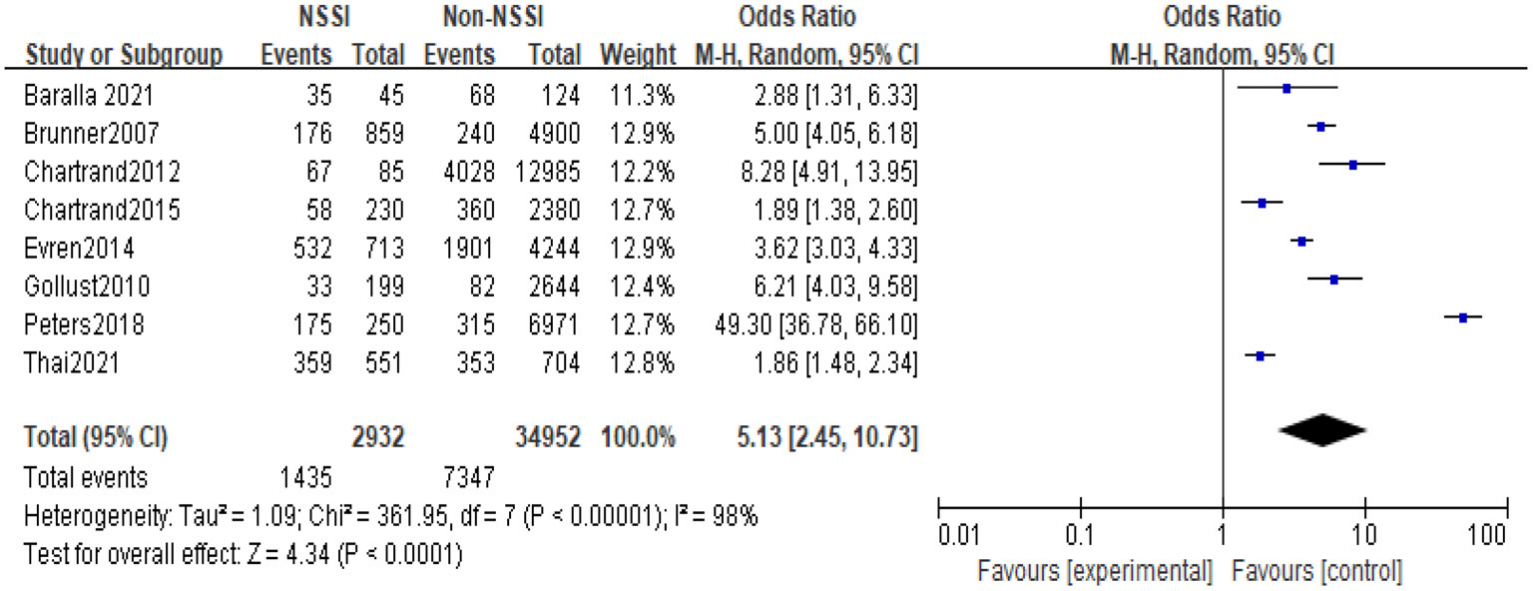
Results of subgroup analysis in the cross-sectional group.

**Figure 8:**
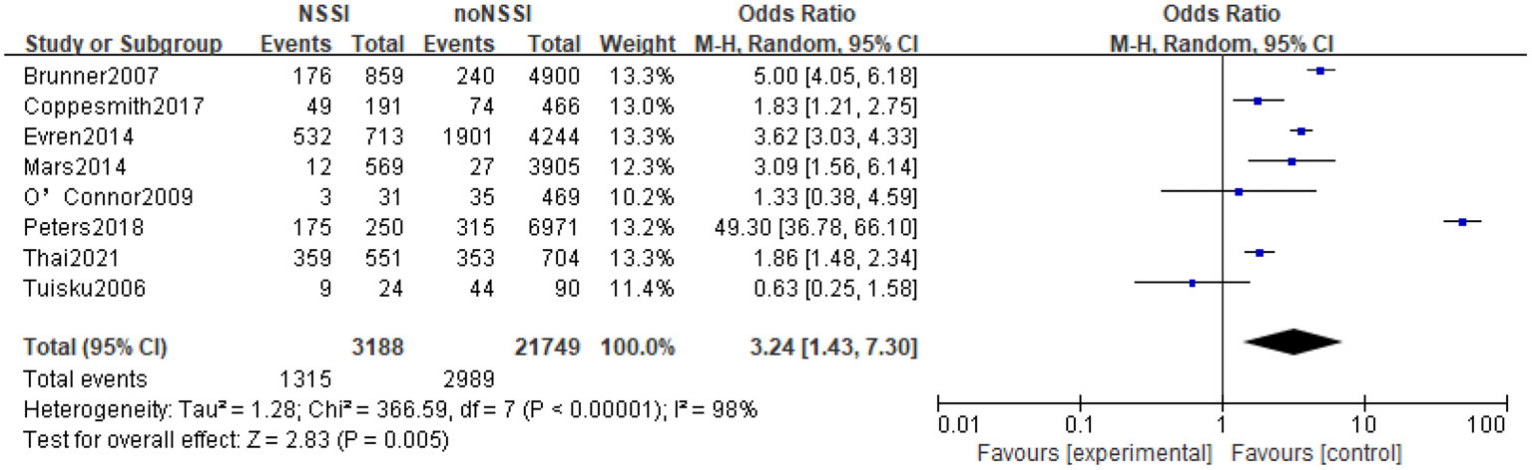
Results of subgroup analysis in the adolescent group.

**Figure 9:**
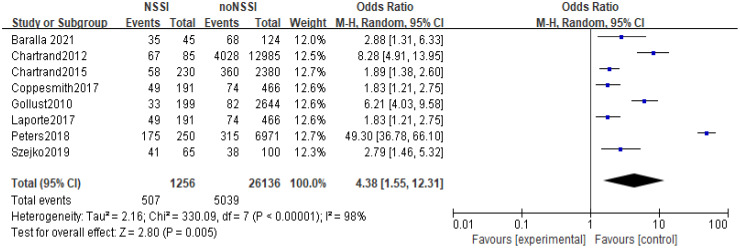
Results of subgroup analysis in the general group.

The 14 original studies of the relationship between anxiety and NSSI were categorized by study type into cross-sectional study groups (n=8) and longitudinal study groups (n=6) and by study sample into adolescent groups (n=6) and general groups (n=8) respectively. Subgroup analyses showed heterogeneity between studies in the longitudinal group (I^2^ = 72%), with a higher proportion of anxiety in the NSSI group than in the control group [OR=2.20, 95% CI (1.29, 3.76), p<0.01] (**Figure 6**); heterogeneity between studies in the cross-sectional group was largely unchanged (I^2^ = 98), and the proportion of anxiety in the NSSI group still had a higher proportion of anxiety than the control group [OR=5.13, 95% CI (2.45, 10.73), p<0.01] (**Figure 7**), suggesting that the cross-sectional study results may have overestimated the effect of anxiety on NSSI. Another subgroup analyses showed heterogeneity between studies in the adolescent group (I^2^ = 91%), with a higher proportion of anxiety in the NSSI group(39.72%) than in the control group(18.17%)[OR=2.45, 95% CI (1.55, 3.89), p<0.01] (**Figure 8**); heterogeneity between studies in the general group was largely unchanged (I^2^ = 98%), and the proportion of anxiety in the NSSI group(40.37%) still had a higher proportion of anxiety than the control group(19.28%)[OR=4.38, 95% CI (1.55, 12.31), p<0.01] (**Figure 9**), suggesting that the adolescent groups was slightly lower effect of anxiety on NSSI.

### 3.5 Sensitivity analysis

Heterogeneity between the NSSI and non-NSSI groups was more pronounced. Except for the exclusion of Peters2018, the heterogeneity decreased (p<0.01, I^2^ = 88%), and after the remaining included literatures were sequentially excluded from the unthinking studies, there was no change in the heterogeneity of the remaining included studies combined (p<0.01, I^2^ = 97%), which suggests that the meta-analysis is more stable and the results are more reliable.

### 3.6 Evaluation of publication bias

**Figure 10:**
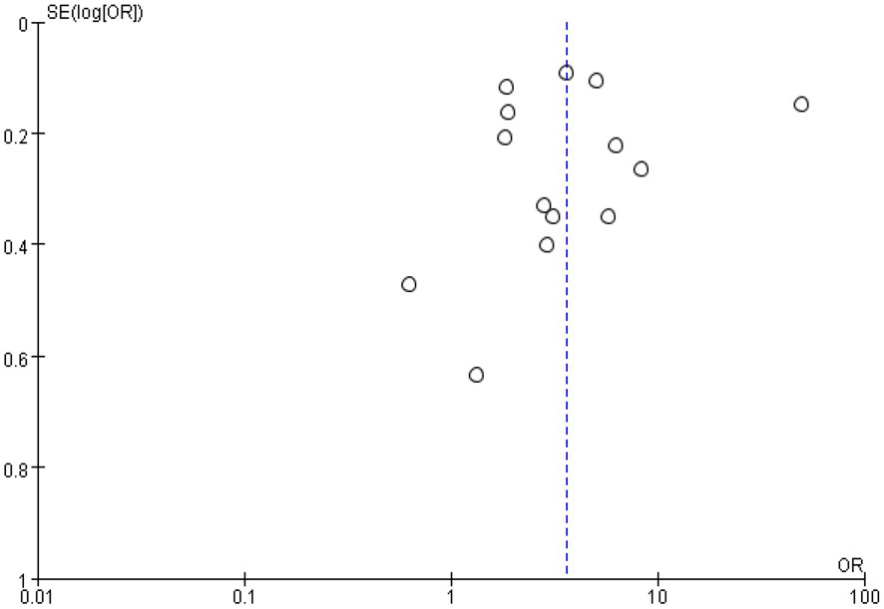
Funnel plot of the relationship between anxiety and NSSI.

Since the study of the relationship between anxiety and NSSI was a comprehensive analysis of 14 previous empirical studies on the subject, a funnel plot was made to analyze the literature for the presence of publication bias. The results showed that the funnel plot was roughly symmetrical (**Figure 10**), suggesting that the possibility of publication bias was low.

## 4 Discussion

In recent years, the prevalence of NSSI has tended to increase significantly, and the increase in NSSI was particularly pronounced during the New Crown pandemic in 2019 (42), and there has been an increasing focus on NSSI-related risk studies, with some evidence of a close relationship between mood and NSSI (43–45). However, studies on the relationship between anxiety and NSSI are still relatively limited, with few in-depth studies devoted to the correlation between anxiety and NSSI, and, there is no comprehensive quantitative analysis assessing the existing studies on the relationship between anxiety and NSSI. The present study included 14 studies (including 6 longitudinal studies and 8 cross-sectional studies) involving 44,064 study participants, aged between 5 and 68 years (most of whom were adolescents), including representative populations in communities, hospitals, schools, and prisons in 11 countries in Europe, North America, Asia, and Oceania. The included literature was evaluated by the AHRQ quality assessment, and the scores were all above 6, which is a medium-high level. The results showed that the proportion of anxiety in the NSSI group was higher than that in the non-NSSI group, and the random-effects model showed that anxiety was significantly associated with NSSI (OR=3.60, 95% Cl =2.08-6.22, p<0.01), which is in line with the results of most of the previous studies. Subgroup analysis results, both cross-sectional studies (OR=5.13, 95%Cl =2.45~10.73, p<0.01) and longitudinal studies (OR=2.20, 95%Cl =1.29~3.76, p<0.01) showed that anxiety was positively correlated to NSSI with a statistically significant difference; although cross-sectional studies cannot infer the causality between anxiety and NSSI, longitudinal studies suggest that anxiety was a risk factor for NSSI; Sensitivity analyses showed that Meta-analysis had better stability and more reliable results. In summary, anxiety may be a risk factor for NSSI.

Previous studies have shown that high anxiety state contributes to the occurrence of NSSI, mainly due to the fact that individuals with high anxiety levels are more sensitive to stress and are more likely to be in a tense emotional state (46), and that high anxiety is a significant causative factor for the occurrence of NSSI (47). According to the theory of emotion regulation model, people may be able to regulate negative emotions by implementing NSSI behaviors that distract anxiety (48). A survey of NSSI among Chinese college students indicated that the interlocking mediating effects of stressful life events and negative emotions play an important role in NSSI (49). However, most people currently have insufficient knowledge about NSSI behaviors and their correlation with anxiety to properly understand NSSI behaviors and predictive risk factors, thus missing the opportunity to identify and intervene in NSSI and subsequent suicide risk at an early stage. The present Meta-analysis, the first comprehensive and systematic evaluation of the correlation between anxiety and NSSI, provides a basis for a better understanding of the phenomenon of NSSI and for further research.

Additionally, this study suggests that meta-analysis using knowledge graphs is an effective approach as we can semantically query relevant literature for precise analysis and reason about semantically annotated data based on medical terminology (i.e., SNOMED CT in this study). As we have discussed above, the main advantage of the knowledge graph approach is that Knowledge graphs contain the medical domain knowledge with the standard clinical terminology such as SNOMED CT. They can be used for reasoning over the concept hierarchy to exhaust all of the sub-concept labels about anxiety. For example, compared to existing meta-analyses on NSSI risk factors that used tradition (e.g., PMC8938878), PMC 8938878 retrieved 13152 articles, of which 2069 were duplicate ones. Using the knowledge graph approach, we retrieved 448 articles, and there were no duplicates except for one article with a different title.

Thus, that approach is much more efficient than using traditional retrieval method (i.e., keyword search), because it is time consuming and almost impossible for a human to exhaust all keywords and their synonyms of the sub-concepts concerning anxiety.

## 5 Limitations and future implications

For this Meta-analysis, although the total sample size was adequate, the effects of confounding factors on the results were mostly controlled for, and the combined results had good reliability. However, there are some limitations. First, due to the language limitation of the knowledge graph query, only publicly published English literature was collected in this study, and language bias could not be excluded. However, the funnel plot for testing publication bias showed that even if publication bias existed, it was not obvious in this study, so our findings are somewhat persuasive; second, different studies assessed anxiety in different ways, and at the same time, there is a lack of valid research on the correlation between different types of anxiety and the NSSI, although the different types of anxiety may not be independently present and may be coexisting; and different studies on the NSSI assessment tools also vary. All of these may have some impact on the findings; third, the original studies included different subjects, on the one hand, there are cultural background and ethnic differences in the occurrence of NSSI (50, 51), and on the other hand, there are large differences between the general population and the psychiatric outpatient population, which may affect the findings; fourth, the limitations of the types of included studies, especially cross-sectional studies, can only find the correlation between anxiety and NSSI correlation, limiting the inference of causality, which needs to be confirmed by more high-quality clinical studies in the future.

## 6 Conclusions

Despite the limitations of this study, it fills a gap in the systematic evaluation of the relationship between anxiety and NSSI and provides quantitative support for existing theories. Given that the results of the study support that anxiety is significantly associated with NSSI, it suggests that the population can be targeted with knowledge and skills of emotion management to reduce anxiety sensitivity and minimize NSSI and subsequent suicidality.

Furthermore, we show that using knowledge graphs is an effective approach to retrieve literature for meta-analysis.

## Data Availability

The datasets presented in this study can be found in online repositories. The names of the repository/repositories and accession number(s) can be found below: https://wasp.cs.vu.nl/NSSI/data/.
